# *Drosophila* Dynein Intermediate Chain Gene, *Dic61B*, Is Required for Spermatogenesis

**DOI:** 10.1371/journal.pone.0027822

**Published:** 2011-12-01

**Authors:** Roshan Fatima

**Affiliations:** Cytogenetics Laboratory, Department of Zoology, Banaras Hindu University, Varanasi, India; University of Hyderabad, India

## Abstract

This study reports the identification and characterization of a novel gene, *Dic61B*, required for male fertility in *Drosophila*. Complementation mapping of a novel male sterile mutation, *ms21*, isolated in our lab revealed it to be allelic to *CG7051* at 61B1 cytogenetic region, since two *piggyBac* insertion alleles, *CG7051^c05439^* and *CG7051^f07138^* failed to complement. *CG7051* putatively encodes a Dynein intermediate chain. All three mutants, *ms21*, *CG7051^c05439^* and *CG7051^f07138^*, exhibited absolute recessive male sterility with abnormally coiled sperm axonemes causing faulty sperm individualization as revealed by Phalloidin staining in Don Juan-GFP background. Sequencing of PCR amplicons uncovered two point mutations in *ms21* allele and confirmed the *piggyBac* insertions in *CG7051^c05439^* and *CG7051^f07138^* alleles to be in 5′UTR and 4^th^ exon of *CG7051* respectively, excision of which reverted the male sterility. *In situ* hybridization to polytene chromosomes demonstrated *CG7051* to be a single copy gene. RT-PCR of testis RNA revealed defective splicing of the *CG7051* transcripts in mutants. Interestingly, expression of cytoplasmic dynein intermediate chain, α, β, γ tubulins and α-spectrin was normal in mutants while ultra structural studies revealed defects in the assembly of sperm axonemes. Bioinformatics further highlighted the homology of *CG7051* to axonemal dynein intermediate chain of various organisms, including DNAI1 of humans, mutations in which lead to male sterility due to immotile sperms. Based on these observations we conclude that *CG7051* encodes a novel axonemal dynein intermediate chain essential for male fertility in *Drosophila* and rename it as *Dic61B*. This is the first axonemal *Dic* gene of *Drosophila* to be characterized at molecular level and shown to be required for spermatogenesis.

## Introduction

Spermatogenesis is highly conserved across animal taxa and involves a series of orchestrated steps of cell division and morphogenesis that lead to the production of a large number of sperms. *Drosophila* spermatogenesis is studied extensively and requires a large number of spatially and temporally synchronized events and processes [1]–[3]. Thus, unsurprisingly, mutations in more than 10% (∼1500 genes) of the protein coding genes in *Drosophila* genome have already been identified to be involved in male fertility [4].

During our course of studies on the functional analysis of a non-coding, developmentally active and heat shock inducible gene of *Drosophila*, *hsrω*, it was reported that a P-transposon insertion allele of this gene, *hsrω^05241^*, was responsible for recessive male sterility [5]. However, further studies on the *hsrω^05241^* allele revealed that the recessive male sterility, initially ascribed to the mutation in *hsrω* gene, was actually due to a background second site mutation, which was named as *ms^21^* [6].

In the present communication, we report the cytogenetic mapping of the *ms^21^* mutation and show that it is allelic to *CG7051* gene located at 61B1 cytogenetic region, which putatively codes for a Dynein intermediate chain (Dic) protein. We further report the molecular genetic characterization of *CG7051* gene and three of its mutant alleles, *ms^21^*, *PBac(PB)CG7051^c05439^* and *PBac(WH)CG7051^f07138^* and show that the *CG7051* gene is required for male fertility in *Drosophila* and rename it as *Dic61B*.

## Results

### Complementation mapping of the *ms^21^* mutation

The recessive male sterile *ms^21^* mutation was maintained with the *TM6B* balancer [7], which established its linkage to chromosome 3 [6]. To map the cytological location of the *ms^21^* mutation, a series of overlapping, molecularly defined Drosdel and Exelixis deficiencies spanning the entire third chromosome were used. Two deficiencies, *Df(3L)ED201* and *Df(3L)Exel6083*, with the break points 61B1-61C1 and 61A6-61B2, respectively, failed to complement the male sterile phenotype of *ms^21^* and accordingly it was mapped to the 61B1-61B2 interval, at the tip of left arm of chromosome 3. Complementation analysis with transposon insertion lines of this region revealed that two *piggyBac* insertion alleles of Exelixis collection [8], viz., *PBac(PB)CG7051^c05439^* and *PBac(WH)CG7051^f07138^*, failed to complement *ms^21^* and the above mentioned deficiencies. These two alleles are annotated to carry *piggyBac* transposons in the 5′UTR and 4^th^ exon of the *CG7051* gene respectively (http://www.flybase.org). Homozygous males of these two alleles and their hetero-allelic combinations were found to be male sterile, while the females were fertile. It may be noted that although the *PBac(WH)CG7051^f07138^* allele is listed on the Flybase (http://www.flybase.org) as viable and fertile, this insertion was found to result in absolute recessive male sterility. Various deficiency and transposon insertion lines used in this study are listed in Supplementary Tables S1 and S2, respectively.

### *CG7051* putatively encodes a Dynein Intermediate Chain (Dic) gene

The *CG7051* gene identified to be responsible for the *ms^21^* mutation is 2.911 kb in size, located on the minus strand (3L: 149679..152589, accession no: NT_037436) and is annotated to code for a component of cytoplasmic dynein based on sequence homology (http://www.flybase.org). Further, it is predicted to encode two polypeptides of 87 kD and 77.9 kD molecular weight with conserved WD40 repeat domains in the C terminal.

It may be noted that Dyneins are minus end directed microtubule based molecular motors which use the energy of ATP hydrolysis to generate movement [9]. These multisubunit complexes consist of heavy chains (∼500 kD), intermediate chains (∼74 kD), light intermediate chains (∼55 kD) and light chains (8–10 kD) and are classified into cytoplasmic dyneins, required for the movement of vesicles, organelles etc [10]–[11] and axonemal dyneins, required for motility of cilia and flagella [12]–[13].

The annotated molecular weight and WD40 repeat domains of *CG7051* encoded polypeptides place this gene in the dynein intermediate chain (Dic) category, as also classified by Goldstein and Gunavardene [14]. Hence, the *CG7051* gene is renamed as *Dic61B* (for Dynein intermediate chain at 61B), following the convention for other fly dynein genes [15]–[16]. The *piggyBac* insertion alleles, *CG7051^c05439^* and *CG7051^f07138^*, are renamed as *Dic61B^c05439^* and *Dic61B^f07138^*, respectively, and *ms^21^* as *Dic61B^ms21^*. For simplicity, these three mutant alleles will be henceforth referred to as *c05439*, *f07138* and *ms21*, respectively.

### *Dic61B* mutants exhibit developmental delay and temperature sensitive lethality

**Figure 1:**
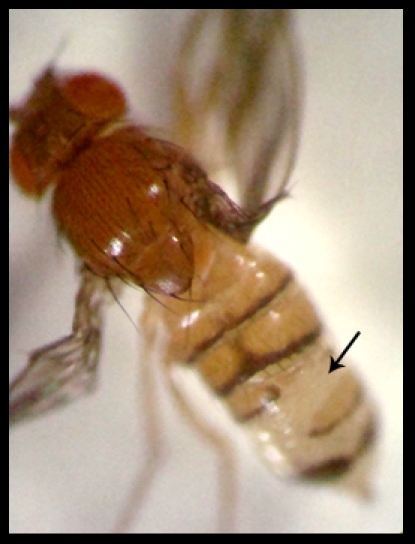
Abdominal tergites are defective in *f07138*. Image showing the abnormally formed abdominal tergites (arrow) in an *f07138* homozygous fly. Wings were deliberately held apart to show the abdomen clearly.

Although all the three mutant alleles under study, *ms21*, *c05439* and *f07138*, are homozygous viable, both the *piggyBac* insertion lines show developmental delay and eclose 24 to 36 hrs later than wild type, at both temperatures tested, 22°C and 25°C. Not only this, about 70% of the homozygous *f07138* flies show moderate to severe defects in the formation of dorsal abdominal tergites (Fig. 1) and ventral sternites (data not shown), while the other two alleles are phenotypically normal.

In view of the reported temperature sensitivity of mutant for *Dic19C*, the unique cytoplasmic Dic gene of *Drosophila* [16], it was examined if the *Dic61B* mutants also exhibited temperature sensitivity. Interestingly, all the three mutants exhibited substantial pupal lethality at 29°C, with only about 18–20% of homozygous and 55–60% of heterozygous pupae eclosing as flies (Table 1), unlike wild type, which showed almost cent percent eclosion at this temperature.

**Table 1:** Percent eclosion of wild type and *Dic61B* mutant pupae at 29°C.

WT	*ms21/TM6B*	*ms21* homozygous	*c05439/TM6B*	*c05439* homozygous	*f07138/TM6B*	*f07138* homozygous
98%(n = 100)	57%(n = 119)	18%(n = 69)	55%(n = 69)	20%(n = 65)	60%(n = 68)	18%(n = 64)

### *Dic61B* mutants produce non-motile and unindividualized sperms

**Figure 2:**
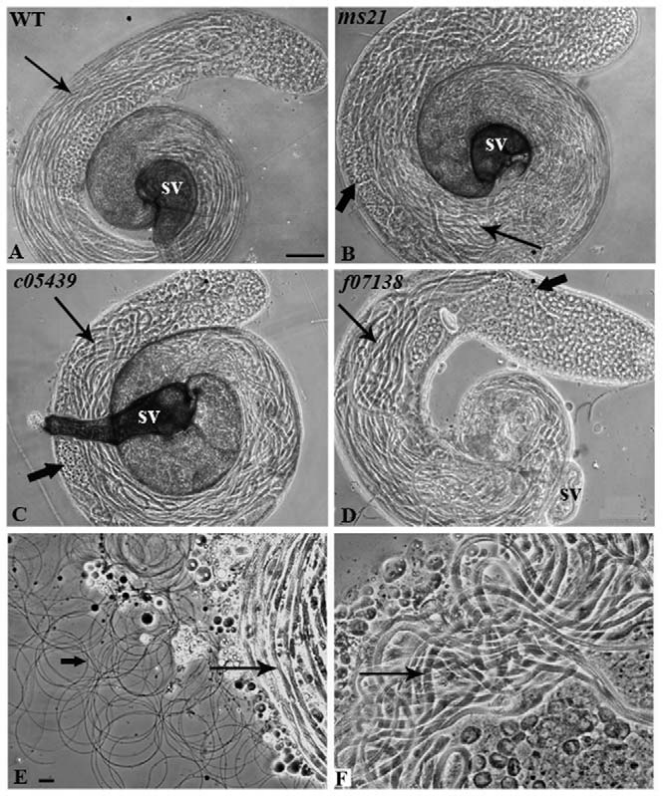
Sperm axonemes are abnormally coiled and remain as non motile bundles in mutants. Phase contrast images of intact (**A–D**) or lightly squashed (**E–F**) testes of wild type (**A, E**), *ms21* (**B, F**), *c05439* (**C**) or *f07138* (**D**) male flies. Note the abnormally coiled sperm tails in mutants (thin arrows in **B–D**), while they are straight in wild type (arrow in **A**). Block arrows point to the misplaced cysts on the convex side of the testis in **B** and **C** and to the spermatid cyst preceding the spermatocyte cysts in **D**. The seminal vesicles (**SV**) are filled with sperms in wild type (**A**) but either filled with debris (**B** and **C**) or are empty (**D**) in the mutants. Motile and individualized sperms (block arrow) together with sperm bundles (arrow) are seen in partially squashed testes from wild type (**E**) but only unindividualized, immotile and bent sperm bundles are seen in testes from *ms21* flies (arrow in **F**). Bar in **A** = 100 µm for **A**–**D**. Bar in **E** = 20 µm, for **E**–**F**.

Phase contrast microscopic examination of testes from the three *Dic61B* mutants revealed that unlike the well ordered arrangement of the various spermatogenetic stages in wild type testes (Fig. 2A), the mutants testes were packed with abnormally bent sperm bundles, making the testes bulge slightly in the middle region and displacing all other cell types from their orderly arrangement (Fig. 2B–D). Further, their seminal vesicles were completely devoid of any sperm and hence, light squashes of mutant testes released only bundles of non-motile sperms (Fig. 2F, only *ms21* data shown) rather than the characteristically motile sperms seen in wild type (Fig. 2E).

### Individualization Complexes assemble normally but show defective progression in the *Dic61B* mutant testes

Mature spermatozoa are produced in *Drosophila* from sperm bundles through a process called sperm individualization, during which Individualization Complexes (ICs) consisting of 64 cones of actin assemble around each immature sperm nucleus and move towards the caudal ends of the sperm tails, removing all cytoplasm and organelles in the form of a waste bag and producing 64 thin, individual sperms wrapped in their own membranes [17]–[19].

**Figure 3:**
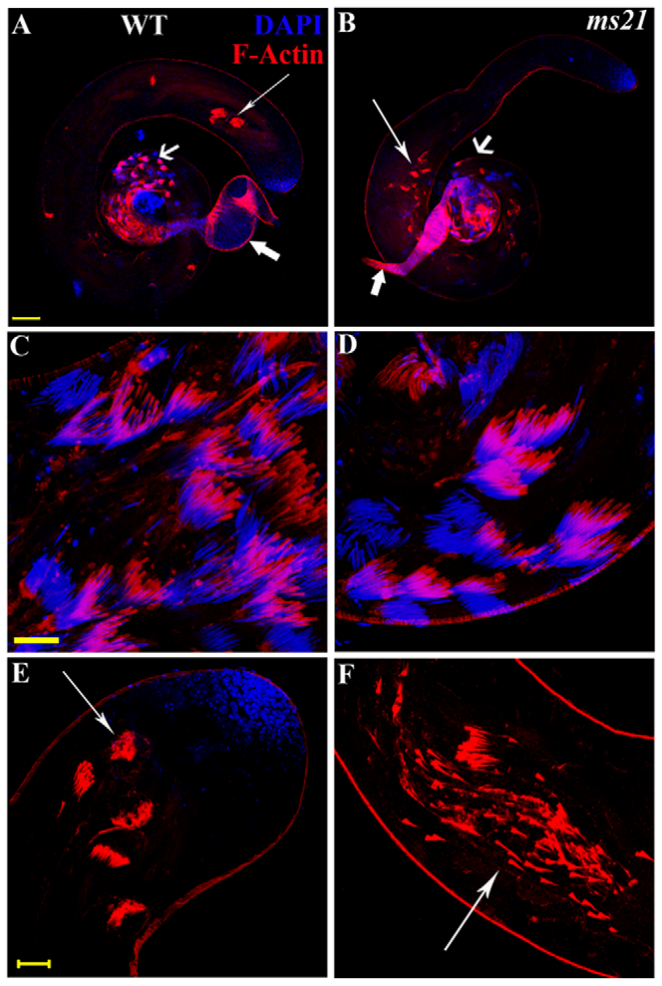
Invidualization of sperms is flawed in mutants. Confocal images of Phalloidin (red) and DAPI (blue) stained testes from wild type (**A**, **C**, **E**) and *ms21* mutant (**B**, **D**, **F**) flies showing the organization of individualization complexes (ICs). **A–B,** ICs assembled in the basal region of wild type and *ms21* testes (short thin arrows in **A** and **B**, respectively). The ICs move synchronously towards the apical end where waste bags are formed in wild type testis but in *ms21*, the ICs manage to travel only till the middle region of testis (long arrows in **A** and **B**, respectively). Seminal vesicles in wild type are swollen due to storage of sperms but are very thin in *ms21*, being completely devoid of sperms (block arrows in **A** and **B**, respectively). **C–D,** Higher magnification images of ICs assembled normally in the basal region of wild type and *ms21* testes respectively. **E–F,** Higher magnification images of waste bags formed at the apical end of testis on completion of individualization in wild type and the progressing ICs disrupted en-route to the apex in *ms21* (arrows in **E** and **F** respectively). Bars in **A, C** and **E** represent 100, 20 and 10 µm, respectively.

Wild type testes stained with TRITC-conjugated Phalloidin and DAPI showed the presence of well-organized ICs, with the actin cones moving synchronously away from the DAPI stained sperm heads, from the base of the testis (Fig. 3A,C) towards the apical end where waste bags were seen (Fig. 3E). Interestingly, well-organized ICs were formed in mutants also (Fig. 3B,D), but the actin cones were disrupted soon after commencing their journey towards the caudal end of sperm tails and degrade prematurely in the middle region of testis, before the sperms are individualized (Fig. 3F; only *ms21* data is shown for all cytochemical studies, the other two alleles are essentially similar to *ms21* in these aspects).

**Figure 4:**
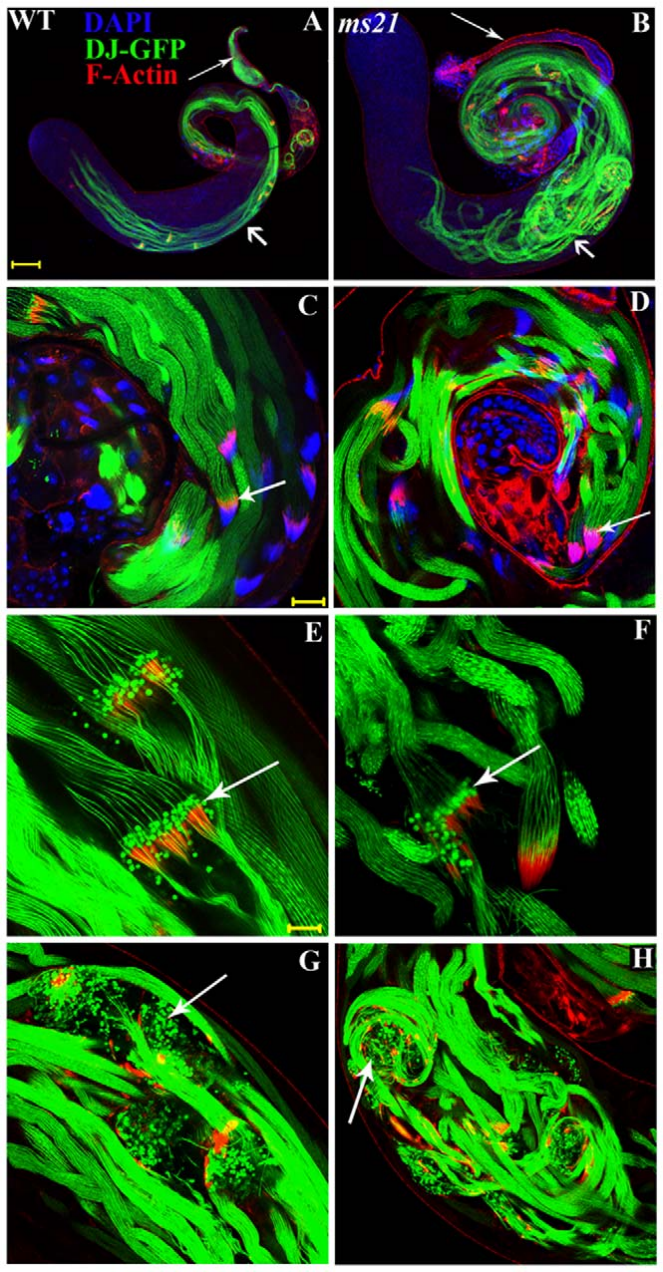
Abnormally bent sperm axonemes obstruct the movement of ICs in mutants. Confocal micrographs of testes from *DJ-GFP* (**A, C, E** and **G**) and *ms21 DJ-GFP* (**B, D, F** and **H**) homozygous males showing sperm axonemes labeled with Don Juan-GFP protein (green), phalloidin stained ICs (red) and DAPI stained chromatin (blue). Sperm axonemes in wild type are straight (**A, C, E** and **G**, short arrow in **A**) but those in *ms21* are bent and convoluted (**B, D** and **H**, short arrow in **B**). Long arrows in **A** and **B** indicate the seminal vesicles, filled with sperms in wild type and empty in *ms21*, respectively. Arrows in **C** and **D** show the normally assembled ICs around the sperm heads in the basal coil of wild type and *ms21* testis, respectively. Arrows in **E–F** show progressed ICs separating the bundles of sperms into individual ones in wild type and *ms21* respectively. Note that pearl like structures of DJ-GFP colocalize with the actin cones of ICs. Arrows in **G** and **H** show the waste bags formed near the apical region in wild type and disrupted ICs in *ms21*, respectively, since the ICs of *ms21* get entangled in its abnormally coiled sperm tails. Bar in **A** is 100 µm (for **A–B**), bar in **C** is 20 µm for **C–D** and **G–H**. Bar in **E** is 10 µm, for **E–F**.

Don Juan-GFP (DJ-GFP), a sperm specific protein, which decorates elongated sperm bundles and mature spermatozoa [20] and also colocalizes with the actin cones of individualization complexes [21], was genetically recombined with *ms21* allele for a better analysis of the defective individualization observed in mutants. Phalloidin staining of *ms21-DJ-GFP* recombinant line revealed that the abnormally bent sperm tails of the *ms21* caused obstruction to the progressing actin cones (Fig. 4 B, H), resulting in faulty progression of normally assembled individualization complexes (Fig. 4 D, F).

### Molecular characterization of lesions in *Dic61B* gene in the mutants

**Figure 5:**
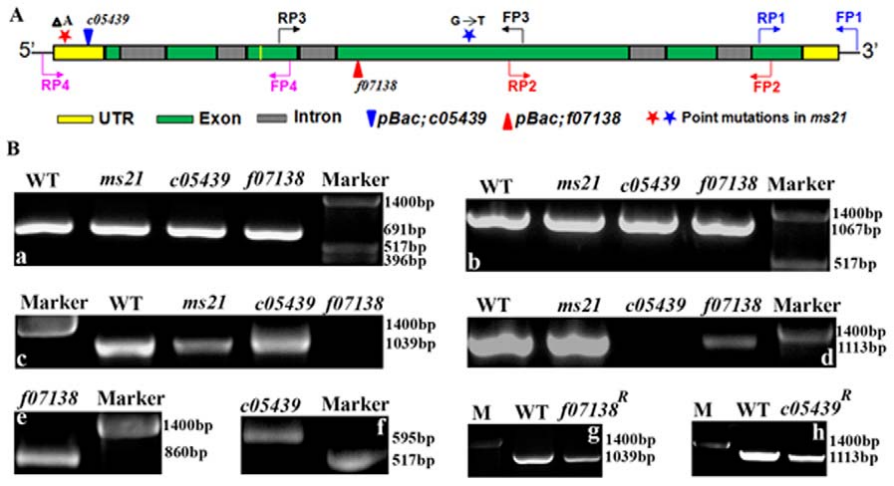
PCR analysis of *Dic61B* gene in wild type and mutants. **A.** Schematic of *Dic61B* gene and primer pairs used for PCR analysis (primer pair numbers are in descending order in 5′ to 3′ direction of the gene). *piggyBac* 5′ and 3′ end specific primers were also used (not shown). **B a–d,** PCR amplicons obtained with primer pairs P1–P4, respectively, with genomic DNA of wild type and mutant lines (indicated on top of the PCR lanes). Note that all the three mutants generated amplicons similar to wild type with the P1 and P2 primer pairs (**a–b**), whereas the primer pairs 3 and 4 did not produce any amplicons with *f07138* and *c05439* genomic DNAs (**c–d**), respectively, due to the presence of *piggyBac* elements in these regions (see panel A). The *ms21* genomic DNA produced amplicons similar to wild type with all the four primer sets (**a–d**). **e–f,** amplicons obtained with a combination of gene specific and *pBac* end specific primers with *f07138* (PB5′-FP3) and *c05439* (PB3′-FP4) genomic DNAs, respectively. **g–h,** amplicons of identical size obtained with genomic DNA of wild type and revertant flies (*f07138^R^* and *c05439^R^*) with primer pairs P3 and P4, respectively. *pUC12* DNA digested with Hinf I was used as the marker in all cases.

To identify the lesions associated with the *Dic61B* gene in three mutant alleles under study, four overlapping primer sets, P1 to P4, spanning the entire gene, including a region of −448 bp upstream to 397 bp downstream of the gene were designed for PCR analysis (see Fig. 5A and Materials and methods). Besides, *piggyBac* specific primers were also used in combination with gene specific primers.

Surprisingly, PCR amplicons generated with the *ms21* genomic DNA with each of the four primer sets were identical in size to those generated from the wild type (Oregon R) genomic DNA (Fig. 5B, a–d). The other two alleles, *c05439* and *f07138* are annotated to have *piggyBac* insertions at +144 bp (5′ UTR, − orientation) and +892 bp (fourth exon, + orientation) of the gene, respectively (http://www.flybase.org). Our PCR analysis (Fig. 5B, c–f) and subsequent sequencing of the amplicons (not shown) confirmed the positions and orientations of the *piggyBac* insertions. Further, excision of the *piggyBac* transposons with the help of *piggyBac* specific transposase source reverted the male sterile phenotype of both these alleles and PCR analyses of the revertants with gene specific primers confirmed precise excisions (Fig. 5B, g–h).

### The *ms21* allele carries two point mutations in the *Dic61B* gene

**Figure 6:**
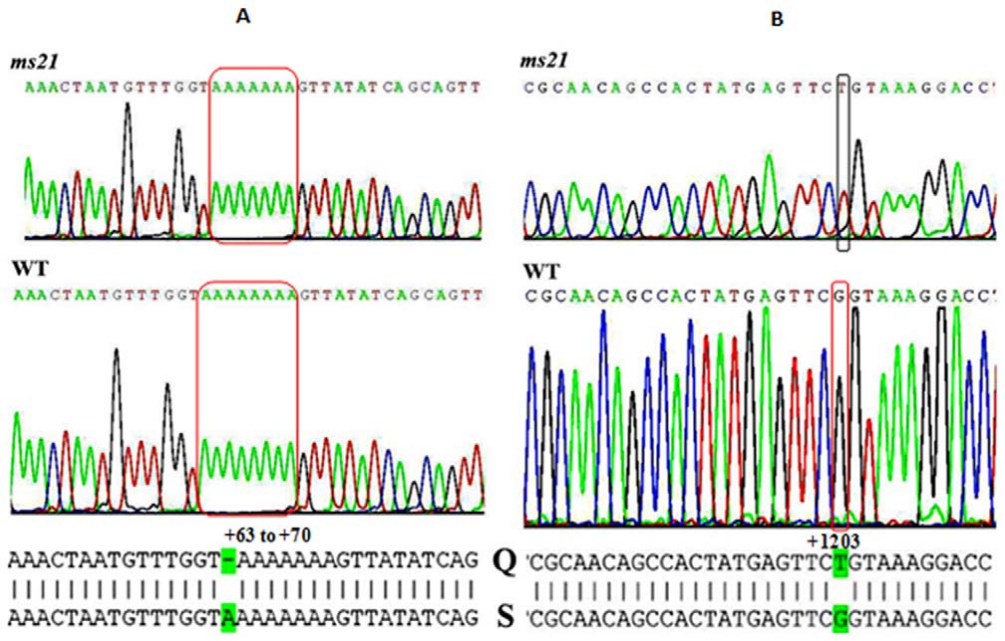
The *Dic61B* gene in *ms21* allele is associated with two point mutations. Chromatogram files of *ms21* and wild type *Dic61B* gene sequences and the corresponding Blast analysis (***ms21*** is query, **Q** and **WT** is subject, **S**). Green highlighted base pairs in Blast analysis in panel **A** show the deletion of one Adenosine in *ms21*, in between positions +63 to +70. In panel **B**, the highlighted base pairs show base substitution (G→T) in *ms21* at position +1203 in the fourth exon, which corresponds to +1204 in wild type (subject), as a single base is deleted in *ms21*, upstream to this position. These changes are marked by rectangles in chromatogram files above them.

Since *ms21* DNA generated amplicons similar to wild type DNA as mentioned above, all the amplicons obtained with wild type and *ms21* DNA were subjected to bidirectional sequencing in order to identify any lesions which could not be resolved through PCR analysis. The *ms21* DNA revealed two base changes (Fig. 6), viz., a deletion of one of the 8 consecutive adenines between +63 to +70 region in the 5′UTR and a synonymous base change, G(1203)T transversion, in exon 4 of the *Dic61B* gene. Interestingly, the sequence for the wild type amplicons obtained by us differed from the *CG7051* (*Dic61B*) sequence available at the Flybase (FBgn0035100) or NCBI (NT_037436.3) databases at two sites, viz., T(1720)A and A(2867)T transversions, of which, the A(2867)T transversion was present in *ms21* allele also. These new sequences have been submitted to the GenBank (vide accession numbers HQ325749, wild type, Oregon R^+^ and HQ456659, *ms21*, respectively).

### Expression of cytoplasmic dynein intermediate chain (Cdic) is not affected in *Dic61B* mutant testes

**Figure 7:**
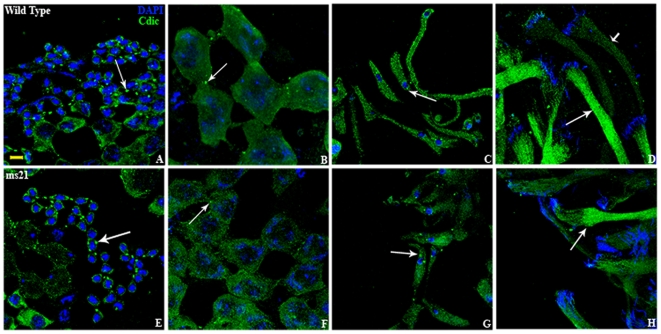
Distribution of Cdic is not affected in the mutants. Confocal images showing various cell types of testes in wild type (**A–D**) and *ms21* mutant (**E–H**) immunostained with anti Cdic antibody, MAB1618 (green) and DAPI stained chromatin (blue). Cytoplasmic distribution of Cdic was observed in hub cells (**A**, **E**), spermatocytes, (**B**, **F**), spermatids (**C**, **G**) and sperm bundles (**D**, **H**). The dot-like structures (arrows in **A**–**C** and **E**–**G**) might represent centrosomes, as Cdic is known to colocalize with centrosomes [53]. Unindividualized sperm bundles showed strong Cdic expression (long arrows in **D** and **H**) whereas those with advanced individualization showed very weak staining (short arrow in **D**).

Since *Dic61B* is annotated to encode a cytoplasmic dynein intermediate chain (http://www.flybase.org), its expression was examined by immuno-fluorescence using anti Cdic antibody, MAB1618, in partially squashed (Fig. 7) as well as intact (not shown) testes, which revealed that the distribution of Dic protein recognized by this antibody was similar in wild type and mutants.

**Figure 8:**
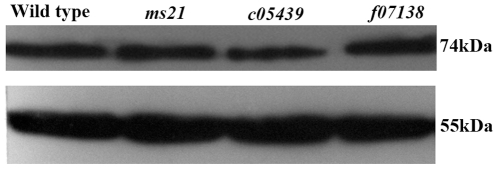
Expression of Cdic in wild type and *Dic61B* mutants is comparable. Western blot showing a comparable 74 kD band detected with the MAB1618 antibody in testes from wild type and the three *Dic61B* mutants (genotypes indicated above the lanes). The lower panel shows tubulin in the same blot as an internal control.

Western blotting analysis of total proteins of adult testes using the same antibody also revealed more or less comparable amounts of a 74 kDa polypeptide in both in wild type and mutants (Fig. 8). It may be noted that the Cdic encoded by the *Dic19C* gene is ∼74 kDa size [22]. Tubulin was used as an internal control to validate equality of total proteins in the different samples (Fig. 8).

### Distribution of Dic61B RNA is not affected but splicing is defective in *Dic61B* mutants

Since the antibody used apparently did not detect the protein product of *Dic61B* gene as described above, the distribution of Dic61B RNA was examined by *in situ* hybridization with an anti-sense riboprobe specific for the gene. Incidentally, the Dic61B RNA distribution was also observed to be similar in wild type and mutant testes (Supplementary Figure S1). The same riboprobe, when used for *in situ* hybridization to polytene chromosomes, revealed the *Dic61B* to be a single copy gene (Supplementary Figure S2).

**Figure 9:**
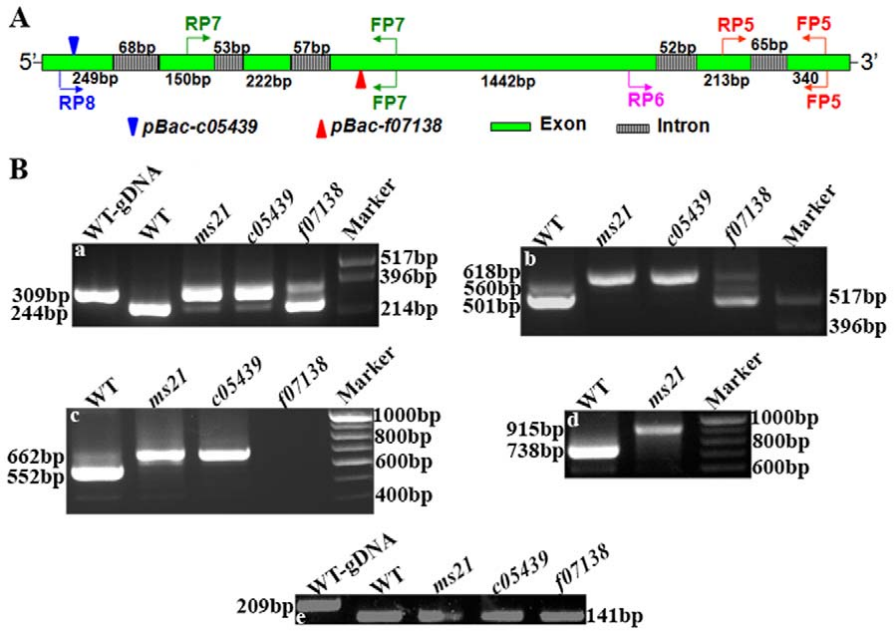
Splicing is defective in *Dic61B* mutants. **A,** Schematic of Dic61B-RA transcript and primer pairs used in RT-PCR analysis. The *Dic61B* gene has six exons (green) and five introns (grey), with their sizes shown below and above them, respectively. Primer pair, FP5-RP5 flanks the last (5^th^) intron, FP5-RP6 pair flanks the 4^th^ and 5^th^ introns together, FP7-RP7 pair flanks the 2^nd^ and 3^rd^ introns together and FP7-RP8 pair flanks the first three introns of the gene. Only one isoform, Dic61B-RA is shown for simplicity. Dic61B-RB differs from RA in sizes of the first intron and second exon, which are of 42 bp and 176 bp, respectively (http://flybase.org). **B (a–d),** RT-PCR analysis of testes RNA from wild type and mutants (indicated on top of respective columns) with the different primer sets shown in panel A. **a,** Amplicons obtained with wild type genomic DNA (first lane) and wild type and mutant testis specific cDNA, with primer set FP5-RP5. Note that wild type cDNA generates an amplicon of smaller size as compared to its genomic DNA, due to removal of intron. The *ms21* and *c05439* cDNAs show unspliced amplicons. In *f07138*, the splicing is more or less comparable to wild type. **b–d,** Amplicons obtained with testis specific cDNA of wild type and mutants, with primer sets, FP5-RP6, FP7-RP7 and FP7-RP8, respectively. Note similar splicing defects in mutants with these primer sets also. No amplicon is seen in *f07138* lane in panel **c** as the FP7-RP7 primer set flanks the *piggyBac* element in this allele (see panel **A**). The *f07138* and *c05439* alleles are not represented in panel **d,** since the primer pair FP7-RP8 flanks the transposons in both these alleles and hence no amplification is seen. **e,** 209 bp amplicon obtained with wild type genomic DNA and 141 bp amplicons obtained with wild type and mutant testis specific cDNAs with primers specific for GPDH internal control.

RT-PCR analysis was carried out using testes RNA of wild type and mutant flies to examine if there is any variation in the expression of Dic61B transcripts. Glycerol-3 phosphate dehydrogenase (GPDH) expression was used as internal control. A primer set specific for GPDH generated amplicons of 209 bp and 141 bp with genomic DNA and cDNA respectively, due to a 68 bp intron in the region of amplification (Fig. 9B, e), which established that there was no genomic DNA contamination in any of the cDNA preparations. The same cDNA preparations were used to analyze transcripts of *Dic61B* gene in wild type and mutants.

The different primer sets used for RT-PCR analysis of *Dic61B* gene are represented in Fig. 9A. This gene has five introns (http://www.flybase.org). The primer set FP5-RP5, flanking the last intron (65 bp) of the gene generated amplicons of 309 bp and 244 bp with wild type genomic DNA and cDNA, respectively, confirming removal of intron (Fig. 9B, a). With *ms21* and *c05439* cDNAs, it generated intense amplicons of 309 bp and very faint amplicons of 244 bp showing that most of the RNA remains unspliced in these mutants (Fig. 9B, a). In *f07138*, the splicing was more or less comparable to wild type, though some unspliced product was also seen (Fig. 9B, a). Splicing defects were also observed in the mutants with the other primer sets, FP5-RP6, FP7-RP7 and FP7-RP8 (Fig. 9B, b–d), which flank the remaining four introns of the gene.

Further, amplicons obtained with RT-PCR of wild type and mutants with FP5-RP5 primer pair were sequenced, which confirmed their specificity for the *Dic61B* gene (data not shown).

### Distribution of various tubulins and spectrin is not affected in *Dic61B* mutant testes

**Figure 10:**
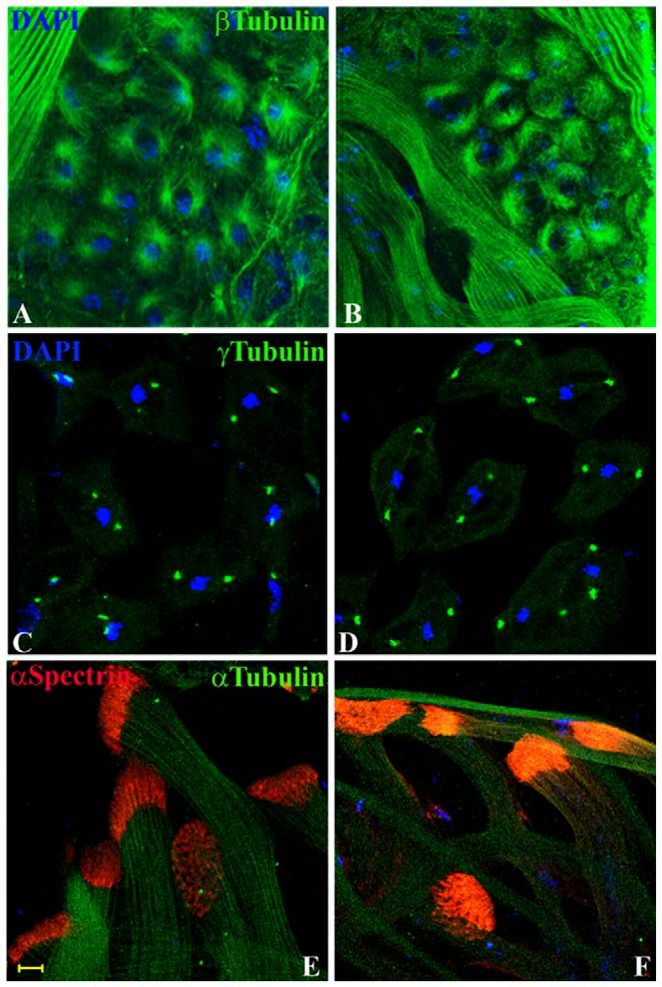
Localization of Tubulins and Spectrin is not affected in the mutants. Confocal images showing comparable organization of asters (green in **A**–**B**, detected with anti β tubulin, E7 antibody), centrosomes (green in **C**–**D**, detected with anti γ tubulin, GTU88 antibody), during meiosis I in spermatocytes and of the elongation cones at the caudal end of sperm tails (red in **E**–**F**, detected with anti α Spectrin 3A9 antibody), in wild type (**A**, **C** and **E**) and *ms21* (**B**, **D** and **F**). Sperm tails are visualized with the help of α tubulin (DM1A antibody) staining in **E–F** (green). DNA is counterstained with DAPI (blue) in all panels.

Since cytoplasmic Dynein is a microtubule motor known to be required for various processes involving microtubule dynamics like centrosome localization, spindle assembly etc [23]–[24], the distribution of various tubulins was examined during spermatogenesis in wild type and mutants. It was observed that the aster formation and the centrosomal cycling during various stages of cell division in wild type and the mutant testes were comparable (Green in Fig. 10A–D). Not only this, the distribution of alpha and beta tubulins in all pre and post-meiotic cell types was also normal (data not shown). Furthermore, the distribution of spectrin, aberrant distribution of which is known to result in abnormally bent sperm axonemes leading to male sterility [25], was also normal in the mutants (Red in Fig. 10F).

### Dic61B shares homology with axonemal Dynein intermediate chain of various organisms

**Figure 11:**
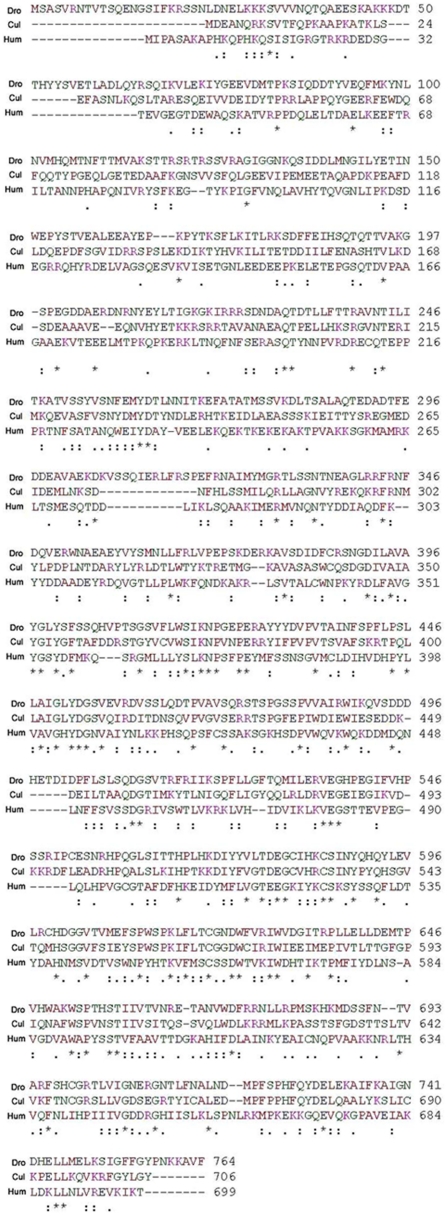
*Dic61B* shares homology with axonemal Dynein intermediate chain of various organisms. Clustal W alignment of the deduced amino acid sequence of Dic61B-PA (Dro) with axonemal dynein intermediate chain inner arm I1 of Culex (Cul) and Axonemal Dynein intermediate Chain 1 (DNAI1) of human (Hum). Note that Dic61B shares higher extent of homology towards the C terminal end with the other two proteins.

Blast search of the predicted amino acid sequence of *Dic61B* gene revealed that it is highly conserved in various *Drosophila* species and that it shares similar extent of homology with both cytoplasmic and axonemal Dynein intermediate chains of *Drosophila* (*Dic19C* and the tandem *Sdic* genes, respectively on the X chromosome, table 2). Compared to these, Dic61B shares higher extent of homology with flagellar/axonemal dynein intermediate chain of various organisms (table 2), including DNAI1 of humans, mutations in which are implicated in male sterility due to immotile sperms [26]. Dic61B shares higher homology towards the C terminal end of the protein with other axonemal Dic proteins, presumably due to the conserved WD40 repeat domains in this region, as observed by Clustal W analysis (Fig. 11).

**Table 2:** Blast analysis of deduced amino acid sequence of *Dic61B* of *Drosophila melanogaster*.

Gene	Accession Number	Blast Score (Bits)	Identities	Similarities	Query coverage
GD13539 *D. simulance*	XP_002083091.1	1427	99%	99%	100%
Axonemal Dynein inner arm i1 *Q. quinquefasciatus*	XP_001870816.1	455	42%	60%	85%
Axonemal Dynein inner arm i1 *A. aegypti*	XP_001652044.1	440	41%	59%	85%
Axonemal Dynein intermediate chain 1 (DNAI1) *H. sapience*	NP_036276.1	147	27%	48%	59%
Cytoplasmic Dic19C *D. melanogaster*	AAF73046.1	127	21%	41%	73%
SDIC1 D. melanogaster	AAF45366	94.3	21%	40%	78%

### Mutations in *Dic61B* affect assembly of sperm axonemes

**Figure 12:**
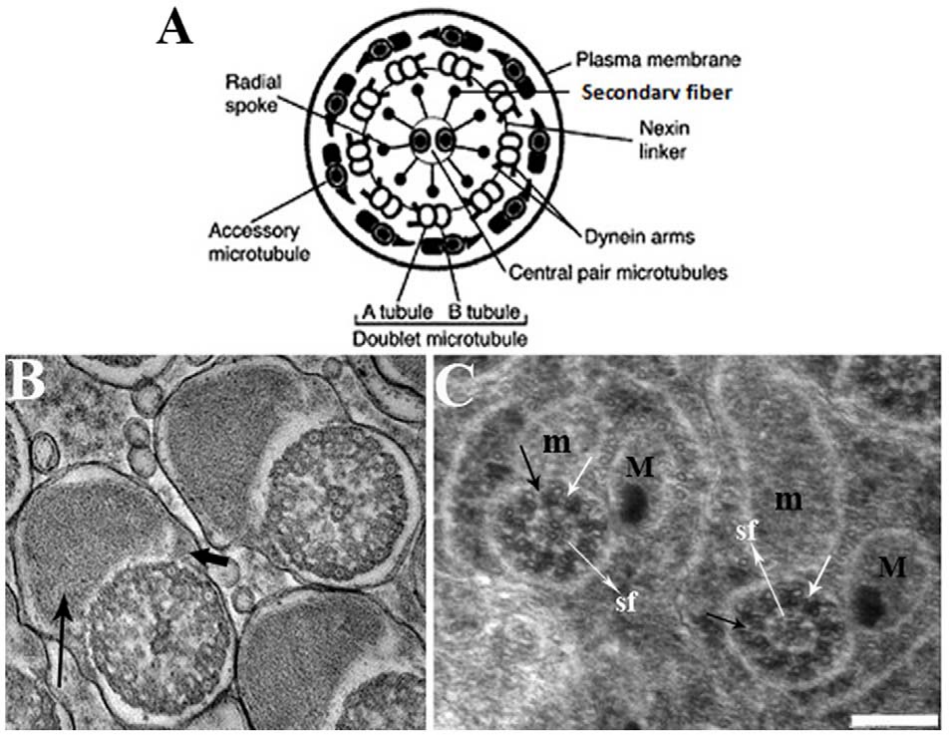
Ultra structure of wild type and *ms21* sperm axonemes. **A,** Schematic of an axial fiber of *Drosophila* sperm, adapted from Nielsen and Raff [72] showing the typical 9+9+2 arrangement of microtubules (see text for details). **B,** Wild type post-individualization axonemes, each wrapped in its own membrane showing the typical 9+9+2 architecture. Each axial fiber in a sperm is associated with two mitochondrial derivatives, a major (M) and a minor (m) one (long and short arrows in B, respectively). **C,** *ms21* axonemes from unindividualized sperm bundle showing various defects: in the axoneme on the left, both the mitochondrial derivatives are of the same size while in the axoneme towards right, m is larger than M, and the two are not properly orientated, unlike in wild type, where M is always larger than m and they are oriented in a particular angle with respect to the axial fiber. The radial spokes and the secondary fibers (sf, long white arrow) in both the axonemes of *ms21* are not uniformly organized. Some of the secondary fibers in the axoneme towards right are enlarged and appear as tubules. Outer and inner dynein arms associated with some of the peripheral doublets in both axonemes are normal (short white arrows) but are reduced in some doublets (black arrows). Since none of the sperm bundle completes individualization in the *ms21* testes, distinct cell membranes between different axonemes were not identifiable. Scale Bar in is 200 nm.

The sperms of *Drosophila* are remarkably long, measuring about 1.8 mm. Each sperm is composed of an axial fiber and two mitochondrial derivatives. The evolutionarily conserved axial fiber consists of a basic 9+2 arrangement of microtubules, though in insects, it has a 9+9+2 arrangement [27]–[32], as a crown of accessory fibers surrounds the peripheral doublets as shown in Fig. 12. A. The central pair and outer doublet microtubules are connected by radial spokes. The outer doublet microtubules are connected to each other by means of nexin links and are associated with outer and inner Dynein arms (Fig. 12A). Preliminary ultra structural studies of wild type (Fig. 12 B) and mutant sperm axonemes revealed various defects in the mutants (Fig. 12 C, data only for *ms21* is shown). The mitochondrial derivatives in the mutants were not properly condensed and in some cases, the minor mitochondrial derivative (m) was larger than the major mitochondrial derivative (M), unlike in wild type where the case is reverse. The radial spokes were not uniformly organized and more than two tubules could be visualized in the central pair, apparently due to the enlargement of some of the secondary fibers. The cell boundaries too were less marked in the mutant sperm bundles. But surprisingly, the dynein arms were not much affected, though they appeared to be reduced in some of the peripheral doublets.

## Discussion

### *Dic61B* encodes a novel Dynein Intermediate Chain protein (Dic61B) of *Drosophila*, essential for spermatogenesis of the fly

This study reports the identification and characterization of a novel Dynein intermediate chain gene of *Drosophila*, *Dic61B*, essential for the spermatogenesis of the fly. The *ms21* mutation [6] was mapped with the help of molecularly defined deficiency and transposon insertion lines, which identified a novel gene, *CG7051*, at 61B1 cytogenetic position to be responsible for this mutation. Though Goldstein and Gunawardene [14] placed the gene at 61A, it is actually located at 61B1 and is a single copy gene as revealed by *in situ* hybridization to polytene chromosomes (Supplementary Figure S2). The *Dic61B* mutants are homozygous viable but exhibit recessive male sterility whereas the females are fertile, revealing that the gene is essential only for the spermatogenesis of the fly. Data on developmental expression of the *CG7051*, as available on the FlyAtlas (http://www.flybase.org) [33], also shows that this gene is prominently expressed in adult testes, with only a low level of expression in other tissues, suggesting an essential role in spermatogenesis. The developmental delay and temperature sensitivity of the *Dic61B* mutant alleles could be related to its low expression in other tissues, though it requires further studies.

### The *piggyBac* insertions in *Dic61B* gene are responsible for the male sterile phenotype associated with *c05439* and *f07138* alleles

The present studies have confirmed that the position and orientation of *piggyBac* transposons in *c05439* and *f07138* are precisely the same as annotated in fly base [8], lending credence to the inverse PCR studies (https://drosophila.med.harvard.edu/). Revertants generated by the excision of *piggyBac* elements in both the mutants were male fertile, confirming the fact that the male sterility associated with these alleles was indeed due to the insertion of *piggyBac* transposons. PCR analyses of revertants further confirmed that the genomic region is repaired well after the excision of the *piggyBac* elements, in agreement with the fact that *piggyBac* is known to exhibit precise excision [8]. The defective formation of tergites and sternites specific to *f07138* allele might be related to the insulator sequence present in the *piggyBac-WH* transposon it carries, causing repression of adjacent genes [34], since the revertant was no more associated with defects.

### Defect associated with *Dic61B* mutants during spermatogenesis is post meiotic

The cell division and differentiation up to the formation of spermatids is normal in the *Dic61B* mutants, with the major defect being a post meiotic lesion with abnormally convoluted sperm bundles. The assembly of individualization complexes (ICs) and the density of actin in the actin cones of ICs in mutants was comparable to wild type. Further, the ICs also commence their journey towards the caudal end of sperm bundles normally, showing that various factors required for these processes are normal in the mutants. But their journey is foiled due to the bent nature of sperm bundles, the track on which they travel, as visualized by Phalloidin staining in DJ-GFP background. Hence individualization *per se* does not appear to be affected in these mutants but it is apparently a secondary consequence of bent and deformed nature of sperm axonemes causing the disruption and degradation of ICs midway through their journey.

In addition, the bent nature of sperm bundles and the consequent retention of the same in testes also appears to be responsible for throwing all other cell types out of their regular arrangement in testes lumen and further causing stress in cyst cells that envelope the sperm bundles, leading to the aggregation of the omega speckles observed in *ms21* testes [6]. Thus the aggregation of omega speckles in cyst cells of *ms21* appears to be the consequence rather than cause of male sterility as had been considered earlier [5]–[6].

### Splicing defects in *ms21* and *c05439* alleles might be related to the mutations in 5′UTR of the *Dic61B* gene

The *ms21* allele carries two point mutations, a single base deletion, ΔA, in a stretch of eight adenosine bases in 5′UTR, and a synonymous base substitution, G→T, in the fourth exon of the *Dic61B* gene, while the *c05439* allele has a *piggyBac* inserted in the 5′ UTR of the gene. RT-PCR analysis revealed global retention of introns in these two alleles, suggesting that the 5′UTR of this gene might be playing a very significant regulatory role in the post transcriptional processing of the Dic61B primary transcripts.

There are several reports of a single base deletion/ substitution in the 5′UTR leading to altered expression of the gene. A single base deletion in 5′UTR of Theiler's virus has been shown to attenuate its nuerovirulence [35]. In another case, one base deletion in 5′UTR of thrombopoietin (TPO) gene is shown to elevate the serum TPO levels leading to familial essential thrombocythemia [36]. Similarly, P element insertions in 5′UTR of Nucleoporin gene, *nup154*, are also known to affect viability and fertility in *Drosophila* [37].

We speculate that the mutations in 5′UTR of *Dic61B* mutants might be affecting the binding of transcription factors and/ or splicing factors. It may be noted that the presence of transcription factors in spliceosome complex is well known [38], and also that UTR sequences can also function as promoter elements in some cases [39]–[40]. In fact, TESS (Transcription Element Search System) software predicted overlapping binding sites for several transcription factors like TBP (TATA binding protein), hunchback etc, at the stretch of eight Adenosine bases, the site of ΔA in *ms21* allele (data not shown). It is also well established that cis acting splicing enhancers and silencers are present in the exonic regions of genes and that purine rich exonic splicing enhancers (ESEs) are very common which are disrupted even by point mutations [41]–[44]. Specifically, adenosine rich elements in 5′UTR are known to affect post transcriptional regulation of gene expression [45]. Moreover, adenosine rich elements in the untranslated leader sequences are also known to play a very significant role in the enhanced transcription of heat shock genes during stress conditions in *Drosophila* [46]–[47]. In fact at least 50% of point mutations causing human diseases are known to affect splicing rather than changing the aminoacid sequence [48] since genomic variations in both coding and noncoding sequences are known to exert deleterious effects on the process of splicing [49]. Hence, it is presumed that the mutations in 5′UTR of *ms21* and *c05439* might be affecting the splicing in these alleles. The synonymous base substitution observed in the fourth exon of *ms21* may also have a role to play in defective splicing observed in this allele. Further studies are needed to understand basis of the inhibition of global splicing of the *Dic61B* gene in these mutants. Since the mutation in *f07138* allele is not in the 5′ UTR, splicing appears to be less affected in this mutant.

### The *Dic61B* mutants are either null or hypomorphic alleles

Present studies confirm that *f07138* is a null allele of *Dic61B* gene, as the *piggyBac* transposon is inserted in the coding region of the gene in this allele, disrupting its open reading frame. The other two alleles exhibit severely defective splicing of Dic61B transcripts, as revealed by RT-PCR analysis, suggesting that there could be a near total absence of a functional protein product in them. The similar temperature sensitivity and male sterile phenotype exhibited by these as the *f07138* null allele correlate well with this fact that these two alleles could also be either null or severely hypomorphic in nature.

### *Dic61B* encodes an axonemal rather than a cytoplasmic Dic

The *Dic61B* gene is annotated to code for a component of cytoplasmic dynein, based on sequence homology (http://www.flybase.org). Other Dynein intermediate chain (Dic) genes reported so far in *Drosophila* include the unique cytoplasmic Dic gene, *Dic19C*, and its adjacent, tandem, axonemal Dic genes, *Sdic1-4* [50]–[51]. The cytoplasmic Dic gene, *Dic19c* is essential in *Drosophila*, though one of its alleles, *sw^1^* (short wing), is homozygous viable and fertile [16]. It exhibits temperature sensitivity and 60% of the ICs (Individualization Complexes) and NBs (Nuclear Bundles) are disrupted in its testes at 25°C [52]. No mutant phenotype has so far been reported for the axonemal *Sdic* genes, except for the information that the *Sdic-GFP* fusion protein is enriched in elongated spermatids and seminal vesicles [51].

The presently characterized *Dic61B* mutants are similar to the cytoplasmic Dic mutant, *sw^1^*, in certain aspects since they also exhibit temperature sensitivity (pupal lethality at 29°C) and disrupted ICs in their testes. These observations, together with the flybase annotation of *CG7051* as a component of cytoplasmic dynein made it a likely candidate for another cytoplasmic Dic of *Drosophila*, which prompted us to examine the expression of Cdic and various tubulins initially.

Studies involving western blotting and immuncytochemical examination with anti Cdic antibody, MAB1618, did not reveal any difference in the levels or distribution of Cdic in any of the mutants under study. Using the same antibody Quintyne and Schroer [53] had shown that Cdic localizes to centrosomes, revealing its involvement in centrosomal function. Not only this, Cdic is known to be a bonafide component of Dynein motor [22], and its mutant condition is expected to affect cell division [23]–[24]. Accordingly, dysfunction of Cdic gene, *Dic19C*, is known to cause lethality in *Drosophila* [16]. But the *Dic61B* mutants under present investigation do not disrupt the localization of various tubulins including asters and centrosomes, or spectrin cytoskeleton and justifiably, do not exhibit lethality. *Dic61B* is predominantly expressed in testes with little or no expression in other tissues as presented in FlyAtlas [33], unlike cytoplasmic Dic which exhibits ubiquitous expression [22]. All these observations suggested that the Dic61B protein is unlikely to be a cytoplasmic Dic, in agreement with a previous report that *Dic19C* encoding cytoplasmic Dic is a unique gene in *Drosophila* [22].

The nature and localization of *Dic61B* gene product appeared to be puzzling initially and tricky to be resolved in the absence of a specific antibody. The similar extent of homology that Dic61B shares with the reported cytoplasmic and axonemal Dic proteins of *Drosophila* (Table 2) caused ambiguity in explicitly categorizing it as either cytoplasmic or axonemal component. It may be noted that the multiple copies of axonemal *Sdic* genes have recently evolved following a fusion of cytoplasmic *Dic19C* gene and its neighboring *annexin* gene [51], due to which they share good extent of homology. But the observation that *Dic61B* is homologous to Axonemal Dynein intermediate chain of various organisms, including DNAI1 of humans implicated in Primary Ciliary Dyskinesia (PCD), associated with infertility due to immotile sperms [26] provided the clue that it might be an axonemal Dic. Various defects pertaining to the sperm axonemes at ultra structural level further strengthened the view that *Dic61B* is required for the axoneme assembly, the absence of which appears to be directly responsible for the bent/convoluted nature of sperm tails, which in turn obstructs the movement of individualization complexes, leading to unindividualized sperms and absolute male sterility.

Flagellar wave form requires radial spokes and central tubules [54]–[55], and defects in these structures are expected to cause bending of axonemes. The *IC138* gene of *Chlamydomonas* encoding a dynein intermediate chain with 7 WD-40 repeat domains is also required for the organization of radial spokes and central pair apparatus [56]. In one of its alleles, BOP5, the flagellar wave form/ bending is affected similar to the bent sperm tail morphology seen in the presently characterized *Dic61B* mutants. Further, all components, except LC7, are assembled in Dynein arms of BOP5, similar to the observation that the Dynein arms are more or less normal in the *Dic61B* mutant sperm axonemes.

Interestingly, Y chromosome deletions affecting the axonemal dynein heavy chains also affect the orientation of mitochondrial derivatives and various other components in sperm axonemes [27], [57]–[58], as observed in presently characterized *Dic61B* mutants. In fact X0 males lacking the entire Y chromosome are homozygous viable and the only phenotype they exhibit is male sterility [59], with various defects in the ultra structure of sperm axonemes, due to which they degenerate before maturation [27]–[30]. Further, mutations in the Dynein light chain, tctex-1 of *Drosophila* are also male sterile, due to defective association of basal body and nucleus and immotile sperms due to the absence of tctex-1 in axonemes [60]–[61].

Since the WD40 domain is required for protein-protein interactions [62], mutations in the WD40 carrying *Dic61B* gene might be leading to defective assembly of the Dynein multisubunit complex and the abnormalities observed in its mutants could be mediated indirectly by the absence of other components, possibly light chains, of the Dynein complex.

### Different axonemal Dyneins are required for different functions

While deletions of fertility factors kl3 and kl5, encoding axonemal dynein heavy chain genes of *Drosophila* [30], Dynein intermediate chain genes of Chlamydomonas [62] and human [26] are known to reduce or eliminate dynein arms specifically, the Dic61B protein appears to be having distinct localization and functions, as the dynein arms are more or less normal but defects are seen in other components of axoneme and it also causes disruption of individualization complexes. In this regard, it may be noted that although the absence of each of the six fertility factors results in male sterility, at ultra structural level, the consequences are not the same. Absence of kl2 fertility factor eliminates an axonemal Dynein heavy chain, but no associated defect in sperm axoneme has been identified in this case [30], [63]. Interestingly, males deficient for kl1 fertility factor produce motile sperms, which are also transferred to the female but they are non functional since they do not enter the seminal receptacle and not used in fertilization [28]. These kl1^−^ flies exhibit a low incidence of disorganized axonemes and occasional defects in the mitochondrial development [28]. A novel WD repeat protein has recently been identified on the Y chromosome, which is suggested to be the kl1 fertility factor [64]. Absence of the ks-2 fertility factor leads to misalignment of the developing axoneme and the mitochondrial derivative, with drastic effects on subsequent spermatid development [30], [63]. Males deficient for h1–h3 or h4–h9 region of Y chromosome displayed disrupted individualization complexes scattered along the spermatid bundle, which was separable from the absence of kl3 or kl5 fertility factors present in this region, which caused only absence of dynein arms but not the disruption of ICs [65]. Hence, the region h1–h9 appeared to carry genetically separable functions, one required for spermatid individualization and the other essential for assembling the axonemal dynein arms [65]. Thus different axonemal dyneins appear to be having distinct, non redundant functions, in agreement with Asai's multi dynein hypothesis [66].

*Dic61B* is the first axonemal Dic gene characterized at molecular level and implicated in male sterility in *Drosophila*. Studies involving transgenic rescue or an over expressing *Dic61B* transgene could be useful by lending further support to our present observations, but we believe that our studies are sufficient to establish the role of the *Dic61B* gene in spermatogenesis of the fly since null allele of this gene is homozygous viable and the only phenotype it exhibits is male sterility. It may further be noted that fertility factors kl2 and kl3 have been identified as 1β and γ axonemal Dynein heavy chain genes [58], but, to the best of our knowledge, the requirement of these for male fertility of the fly has also not been confirmed with the help of transgenic rescue but rather, this has been done mainly with the help of genetic analyses [67]. Hence, we believe that the lack of such studies would not make our present interpretations less accurate.

The presently characterized mutant alleles of novel axonemal dynein intermediate chain protein in *Drosophila* could be useful for further studies on the specific roles of this protein in sperm axoneme development not only in *Drosophila* but in higher organisms as well, as a large number of spermatogenic genes are known to be conserved between fly and humans [68]. Since male infertility is a major problem faced by human population worldwide, there is an absolute need to better understand the genes and proteins that are required for male fertility. In fact much of the information regarding the pathways and regulatory networks involved in male germ cell development in humans has been generated mainly by studying various model organisms. Hence it would definitely be rewarding to gain a better perception of the spermatogenesis of the fly and genes involved in this process so that it could be applied to human beings as well.

## Materials and Methods

### Fly stocks and rearing conditions

Wild type (Oregon R) and mutant flies were reared at 22±1°C on standard food containing agar, maize powder, yeast and sugar. The *ms21/TM6B* mutation was isolated in our lab as a second site mutation associated with the P-transposon insertion allele, *hsrω^05241^*, of the non coding gene, *hsrω* [6]. *PBac(WH)CG7051^f07138^/TM6B* and *PBac(PB)CG7051^c05439^/TM6B* are *piggyBac* insertion mutants obtained from Exelixis stock centre at Harvard University [8]. *DJ-GFP/TM3Sb* is a transgenic line expressing GFP tagged Don Juan protein [20]. This stock was recombined genetically with *ms21* allele through appropriate crosses and maintained as a *DJ-GFP ms21/TM6B* stock. Various deficiency and transposon insertion lines used in this study were obtained from Bloomington or Exelixis stock centre, as listed in tables S1 and S2 (supplementary data).

### Complementation analysis of *ms21* and reversion analysis of *c05439* and *f07138* alleles

Complementation analysis of *ms21* allele was carried out with Drosdel and Exelixis deficiency stocks (tables S1 and S2, supplementary data). Virgin *ms21/TM6B* females were crossed with the males of the various deficiency stocks and the F1 males heterozygous for *ms21* and the deficiency were checked for their fertility status by allowing them to mate with wild type virgin females.

The *piggyBac* transposons in *c05439* and *f07138* lines were excised with the help of *piggyBac* specific transposase source, *Cyo-Tr/Wg^SP-1^* [8], obtained from Bloomington stock centre. Virgin flies from the mutator stocks (*c05439* or *f07138*) were crossed to male flies from the jump starter stock (*Cyo-Tr/Wg^SP-1^*) and orange eyed, curly winged, tubby, F_1_ male flies carrying both the transposase and the *piggyBac* transposon were selected and crossed to *JSK3* (*TM3Sb/TM6B*) virgins and from the next generation, rare white eyed revertant F_2_ flies were selected.

### Phase contrast microscopy of testes

To examine various spermatogenic stages, testes from wild type and different mutant one or two day old adult males were dissected in PBS (130 mM NaCl, 7 mM Na_2_HPO_4_, 3 mM KH_2_PO_4_, pH 7.2) and squashed lightly under the weight of a cover slip. The preparations were examined under phase contrast optics using Nikon E800 microscope.

### Phalloidin staining

Testes were dissected from freshly eclosed or one day old males of the desired genotypes and fixed in 4% paraformaldehyde in 1× PBS for 20 min. Tissues were washed with 0.1% and 1% PBST (Triton X-100 in 1× PBS) for 10 min each successively and incubated with TRITC conjugated Phalloidin (Sigma, 1∶200 dilution in PBS) for 45 min at room temperature in a dark moist chamber. Tissues were washed twice in 0.1% PBST, DNA was counter stained with DAPI (Sigma, USA, 1 µg/ml) and mounted in DABCO (Sigma, USA) for examination by confocal microscopy (Biorad Radiance 2000 or Zeiss LSM 510 Meta).

### Genomic DNA extraction and PCR

Genomic DNA was extracted from 50 flies each of the desired genotypes [69] and used as template for PCR reactions. Sequence for the *Dic61B* gene was obtained from the Flybase and four different primer sets (P1 to P4, as represented in Fig. 5A and Table 3) were designed and obtained from Bioserve (Hyderabad, India). *piggyBac* specific primer sequence was obtained from the iPCR protocols (https://drosophila.med.harvard.edu/).

**Table 3:** Primer pairs used for genomic DNA PCR and for RT-PCR.

Name	Sequence	Position with reference to the *Dic61B* gene sequence	Size (bp)
FP1	5′-AATAACCTTGTTCTCACCCCTG-3′	376 bp to 397 bp downstream of the gene	22
RP1	5′-TTCAACGCCCTGAACGACATG-3′	+2627 to +2647	21
FP2	5′-CTCTAACTCGTCGTATTGGAAGTG-3′	+2660 to +2683	24
RP2	5′-GGAGCATTAAAAATCCAGGCGAG-3′	+1617 to +1639	23
FP3	5′-CGTTACTGGCACATCGTAGTAG-3′	+1654 to +1675	22
RP3	5′-CTTCACTACAATGGTAGCGAAGAG-3′	+637 to +660	24
FP4	5′-TCGTGCTCTTCGCTACCATTG-3′	+645 to +665	21
RP4	5′-TAGATTTTGATGCTGCCGCAAG-3′	−427 to −448	22
FP5	5′-CTCCATAAGAAGCTCGTGGTC-3′	+2661 to +2681	21
RP5	5′-CGCTAACGTCTGGGACTTTC-3′	+2373 to +2392	20
RP6	5′-CATCCACAAGTGCTCAATCA-3′	+2113 to +2132	20
FP7	5′-TCGTAGTTTCGGTTGTCTCG-3′	+986 to +1005	20
RP7	5′-AAAAAGAAGTCCGTGGTGGTT-3′	+345 to +365	21
RP8	5′-CATATGTATTACCAGAAACCAGTGC-3	+90 to +114	25

PCR amplification was carried out with the genomic DNA, in a final volume of 25 µl containing 25 pmoles each of the desired primer pairs and 200 µmoles of each dNTP (Sigma, USA) with 3 U of Taq polymerase (Bangalore Genei, India). The cycling parameters included an initial denaturation of 5 min at 94°C followed by 30 cycles of denaturation at 94°C for 1 min, 1 min annealing at 62°C for primer pair 2, and 66°C for primer pairs 1, 3 and 4, and 60°C for *piggyBac* specific primers, and extension at 72°C for 1 min, except for the last cycle in which the extension time was 5–7 min.

### Sequencing of PCR amplicons

The PCR amplicons were sequenced directly with the help of Applied Biosystems Genetic Analyser 3130. Gel eluted PCR amplicons were processed for cycle sequencing in a 10 µl reaction volume using Applied Biosystems cycle sequencing kit version 3.1 and the fluorescently labeled DNA product was precipitated using Big Dye Terminator Clean up method following manufacturer's instructions and dissolved in Hi-Di (Formamide) and processed further for sequencing. The sequences were analyzed with BLAST service of NCBI.

### RNA isolation and Reverse Transcription-PCR

Total RNA was isolated from wild type and mutant testes with the help of TRIzol reagent (Sigma, USA) following manufacturer's instructions, dissolved in DEPC (Diethyl pyroarbonate, Sigma, USA) treated water and treated with RNAse-free DNAse I (MBI Fermentas, USA, 2 U/µl) at 37°C for 30 min. For reverse transcription (RT) reaction, ∼5 µg of total RNA, 80 pmol of oligo (dT)_17_ primer, 20 U of RNAsin (Amersham), 400 µM each of dNTP mix and 100 U of M-MuLV reverse transcriptase (Fermentas) were taken in a total reaction volume of 20 µl and incubated at 37°C for 1 hr, followed by inactivation at 65°C for 15 min. 1/20^th^ (1 µl) of the reverse transcription product was used for PCR reaction. Primer pairs, FP5-RP5, FP5-RP6, FP7-RP7 and FP7-RP8 (Fig. 9A, Table 3) were used in RT-PCR analysis of *Dic61B* gene. GPDH (internal control) specific primers used are, forward primer 5′-CCA CTG CCG AGG AGG TCA ACT A-3′ and Reverse primer 5′-GCT CAG GGT GAT TGC GTA TGC A-3′.

### Antibodies

Mouse monoclonal antibodies against Cdic (MAB1618, Chemicon, USA), α-tubulin (clone DM1A, Sigma, USA), β-tubulin (E7, DSHB, USA), γ-tubulin (clone GTU88, Sigma, USA), α-spectrin (3A9, DSHB, USA) were used. Mouse secondary antibodies used were Alexa flour 488 conjugated (Molecular probes, USA, 1∶ 200 dilution), CY3 conjugated (Sigma, USA, 1∶ 100 dilution) and Anti mouse-HRP (Bangalore Genie, India).

### Immunostaining of intact/partially squashed testes

For immunostaining, testes from wild type and mutant flies were processed as described earlier [70]. Primary antibodies against Cdic (1∶50 dilution) and β-tubulin (1∶10 dilution) were used in immunostaining of intact testes and those against Cdic (1∶50), α-tubulin (1∶200), γ-tubulin (1∶500) and α-spectrin (1∶50) were used for partially squashed testes. Signal was detected with mouse secondary antibodies in all cases. Chromatin was counterstained with DAPI (1 µg/ml) and imaging was done with confocal microscope.

### Polyacrylamide gel electrophoresis and Western Blotting

Protein samples from wild type and mutants testes were prepared in the sample buffer [69], electrophoresed in denaturing condition in 10% vertical SDS polyacrylamide slab gels using the discontinuous buffer system [71], electrophoretically transferred to PVDF membrane (Millipore, USA) by wet transfer method. The blot was incubated with primary antibody against Cdic (1∶500 dilution) and detected with Anti mouse-HRP secondary antibody (1∶ 1500 dilution) using ECL detection system as per manufacturer's instructions (Pierce, USA). Blot was deprobed by incubating in 100 mM β-mercapto ethanol, 2% SDS, 62.5 mM Tris, pH 6.8 at 50°C for 30 min and reprobed with anti β-tubulin antibody at 1∶ 200 dilution.

### Transmission Electron microscopy

Testes samples were fixed in a mixture of 2% paraformaldehyde and 2.5% glutaraldehyde for 12 hrs in 0.1 M phosphate buffer (pH 7.4) at 4°C and washed in 0.1 M phosphate buffer. The samples were post-fixed with 1% (v/v) osmium tetroxide and dehydrated through a series of acetone gradients, infiltrated with a mixture of propylene oxide and epoxy resin (1∶1) overnight and embedded in pure epoxy resin and dried at 60°C for 72 h. Thin sections (60 nm) were stained with uranyl acetate and lead citrate and were observed in a transmission electron microscope, either JEOL 100 CX at Center for Cellular and Molecular Biology (CCMB, Hyderabad, India) or Morgagni 268D (Fei Company, Netherlands), at All India Institute of Medical Sciences (AIIMS, New Delhi, India).

## Supporting Information

Figure S1 **Distribution of Dic61B RNA is not affected in the mutants.** Confocal images showing distribution of Dic61B1 RNA in the apical region (upper panels) and basal region (lower panels) of wild type and mutant testes by *in situ* hybridization with Dig labeled antisense riboprobe generated using *GHO1827*, a cDNA clone specific for *Dic61B*. Note that the distribution of Di61B RNA (red) is similar in wild type and mutants. Chromatin is counterstained with DAPI (blue).
(DOC)

Figure S2 ***Dic61B*** **is a single copy gene.** An *in situ* hybridization performed to control (Con) and heat shocked (HS) polytene chromosomes with the help of *GHO1827* antisense riboprobe shows a single, specific hybridization signal at the tip of 3L chromosome as shown by black arrows, confirming the specificity of the probe and further revealing *Dic61B* to be a single copy gene. Polytenes in lower magnification are shown in **A** and **B** panels, while higher magnification images of the hybridization signal at the tip of 3L are shown in **C** and **D**, respectively (it may be noted that images shown in **C** and **D** represent different chromosomes, not the higher magnification sections of polytenes shown in **A** and **B**). Interestingly, in control conditions, the hybridization signal appeared to scatter away from the point of origin as seen in **C**, which might represent the RNA being synthesized from the gene, detected by the antisense riboprobe. This was not observed in heat shocked polytenes. Blue arrows in **B** and **D** point to the *hsp83* puff at 63B cytogenetic region. Bars in **A** and **C** represent 100 µm and 10 µm, respectively.
(DOC)

Table S1 List of Drosdel and Exelixis deficiency lines used in complementation analysis of *ms21*.
(DOC)

Table S2 Transposon insertion lines tested for complementation with the *ms21* allele. Stocks obtained from Bloomington and Exelixis stock centers are shown in tables S2A and S2B, respectively.
(DOC)
