# Supplementary figures and images for: Drosophila Dynein Intermediate Chain Gene, Dic61B, Is Required for Spermatogenesis
Source: PLoS One. 2011 Dec 1;6(12):e27822. doi: 10.1371/journal.pone.0027822 (PMC3228723; doi:10.1371/journal.pone.0027822)

**
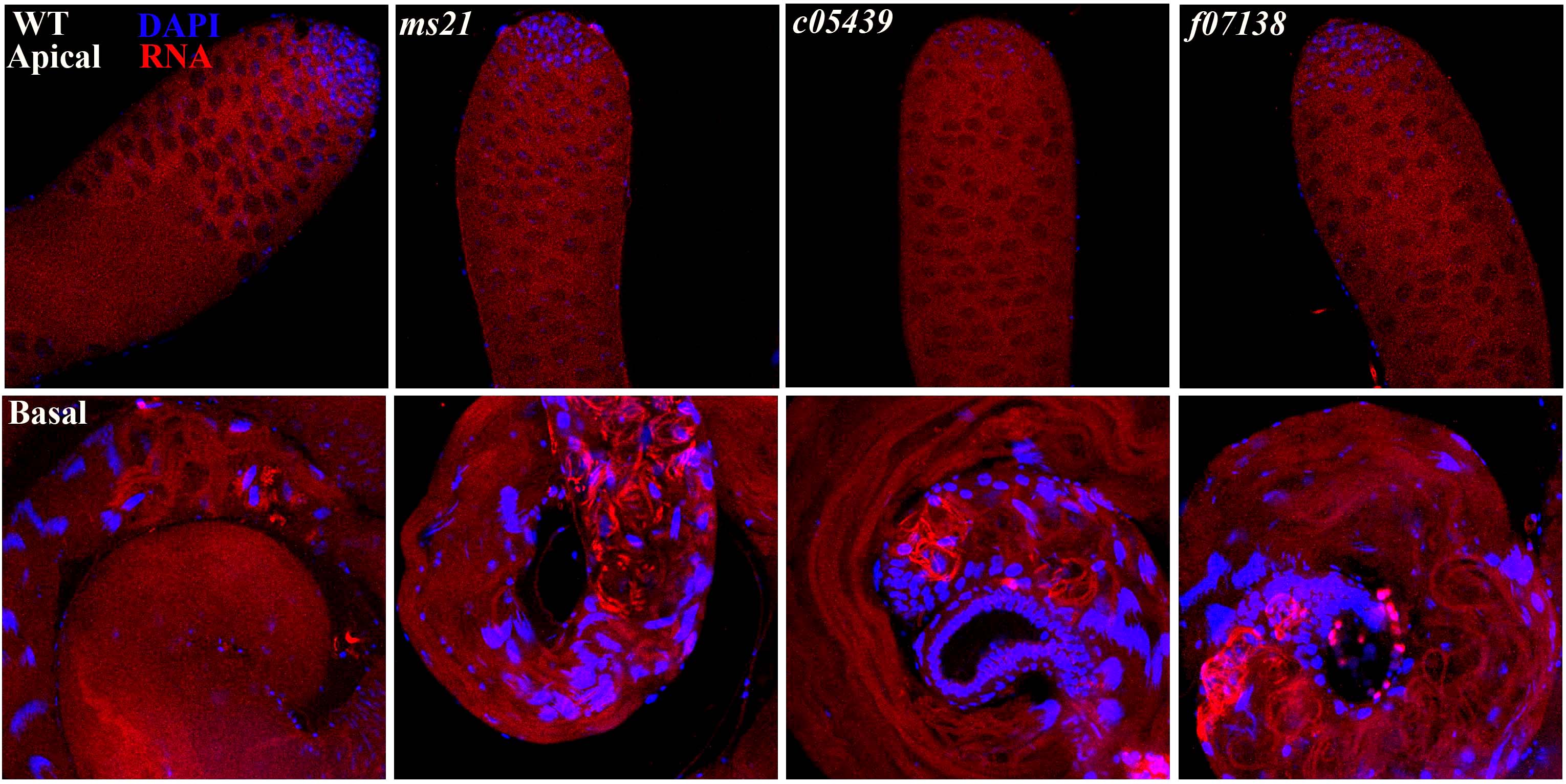
**

**Figure S1**

Supplement: Figure S1 — Distribution of Dic61B RNA is not affected in the mutants. Confocal images showing distribution of Dic61B1 RNA in the apical region (upper panels) and basal region (lower panels) of wild type and mutant testes by in situ hybridization with Dig labeled antisense riboprobe generated using GHO1827, a cDNA clone specific for Dic61B. Note that the distribution of Di61B RNA (red) is similar in wild type and mutants. Chromatin is counterstained with DAPI (blue). (DOC) [file pone.0027822.s001.doc]

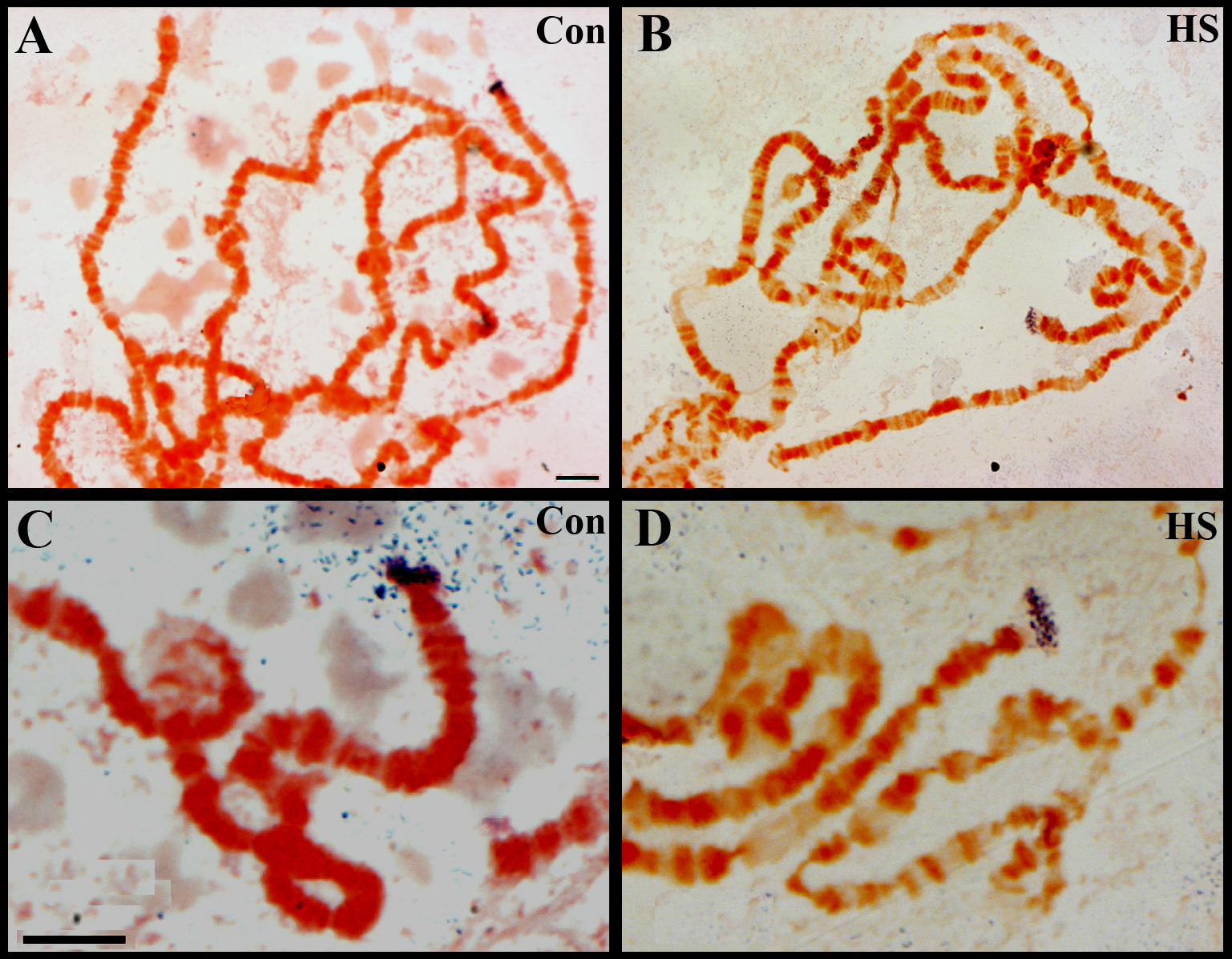


**Figure S2**

Supplement: Figure S2 — Dic61B is a single copy gene. An in situ hybridization performed to control (Con) and heat shocked (HS) polytene chromosomes with the help of GHO1827 antisense riboprobe shows a single, specific hybridization signal at the tip of 3L chromosome as shown by black arrows, confirming the specificity of the probe and further revealing Dic61B to be a single copy gene. Polytenes in lower magnification are shown in A and B panels, while higher magnification images of the hybridization signal at the tip of 3L are shown in C and D, respectively (it may be noted that images shown in C and D represent different chromosomes, not the higher magnification sections of polytenes shown in A and B). Interestingly, in control conditions, the hybridization signal appeared to scatter away from the point of origin as seen in C, which might represent the RNA being synthesized from the gene, detected by the antisense riboprobe. This was not observed in heat shocked polytenes. Blue arrows in B and D point to the hsp83 puff at 63B cytogenetic region. Bars in A and C represent 100 µm and 10 µm, respectively. (DOC) [file pone.0027822.s002.doc]
