# Supplementary material for: Drosophila Dynein Intermediate Chain Gene, Dic61B, Is Required for Spermatogenesis
Source: PLoS One. 2011 Dec 1;6(12):e27822. doi: 10.1371/journal.pone.0027822 (PMC3228723; doi:10.1371/journal.pone.0027822)
Supplement: Table S1 — List of Drosdel and Exelixis deficiency lines used in complementation analysis of ms21. (DOC) [file pone.0027822.s003.doc]

**Table S1**

| **S. No.** | **Symbol** | **Estimated Cytology** | **Genomic sequence coordinates** | **Complemen-tation** |
| --- | --- | --- | --- | --- |
| 1 | Df (3L)ED4079 | 61A5; 61B1 | 3L:40319;131780 | YES |
| **2** | [**Df(3L)Exel6083**](http://flybase.org/.bin/fbidq.html?FBab0038103) | **61A6; 61B2** | **3L:104350;180193** | **NO** |
| **3** | **Df (3L)ED201** | **61B1; 61C1** | **3L:123924;347941** | **NO** |
| 4 | [Df(3L)BSC121](http://flybase.org/.bin/fbidq.html?FBab0043180) | 61B2; 61B2 | 3L:180195;203669 | YES |
| 5 | [Df(3L)BSC128](http://flybase.org/.bin/fbidq.html?FBab0043186) | 61B2; 61B2 | 3L:184799;212410 | YES |
| 6 | [Df(3L)Exel6084](http://flybase.org/.bin/fbidq.html?FBab0038104) | 61B2; 61C1 | 3L:180193;346863 | YES |
| 7 | [Df(3L)BSC125](http://flybase.org/.bin/fbidq.html?FBab0043183) | 61B3; 61B3 | 3L:258029--258031;276882 | YES |
| 8 | [Df(3L)BSC126](http://flybase.org/.bin/fbidq.html?FBab0043184) | 61B3; 61C1 | 3L:271425;327733 | YES |
| 9 | [Df(3L)Exel6084](http://flybase.org/.bin/fbidq.html?FBab0038104) | 61B2; 61C1 | 3L:180193;346863 | YES |
| 10 | [Df(3L)ED208](http://flybase.org/reports/FBab0035326.html) | 63C1;63F5 | 3L:3249148;3893148 | YES |
| 11 | [Df(3L)ED4341](http://flybase.org/reports/FBab0035595.html) | 63F6;64B9 | 3L:3905091;4542236 | YES |
| 12 | [Df(3L)ED210](http://flybase.org/reports/FBab0035327.html) | 64B9;64C13 | 3L:4544234;5348442 | YES |
| 13 | [Df(3L)ED211](http://flybase.org/reports/FBab0035328.html) | 65A9;65B4 | 3L:6211235;6545859 | YES |
| 14 | [Df(3L)ED4408](http://flybase.org/reports/FBab0035657.html) | 66A22;66C5 | 3L:7972207;8292674 | YES |
| 15 | [Df(3L)ED4421](http://flybase.org/reports/FBab0035670.html) | 66D12;67B3 | 3L:8738426;9377175 | YES |
| 16 | [Df(3L)ED4457](http://flybase.org/reports/FBab0035706.html) | 67E2;68A7 | 3L:10357051;11118909 | YES |
| 17 | [Df(3L)ED4470](http://flybase.org/reports/FBab0035719.html) | 68A6;68E1 | 3L:11090089;11826330 | YES |
| 18 | [Df(3L)ED4475](http://flybase.org/reports/FBab0035724.html) | 68C13;69B4 | 3L:11580140;12401701 | YES |
| 19 | [Df(3L)ED4483](http://flybase.org/reports/FBab0035731.html) | 69A5;69D3 | 3L:12270320;12686314 | YES |
| 20 | [Df(3L)ED4486](http://flybase.org/reports/FBab0035734.html) | 69C4;69F6 | 3L:12507519;13025585 | YES |
| 21 | [Df(3L)ED4502](http://flybase.org/reports/FBab0035750.html) | 70A3;70C10 | 3L:13220865;13986651 | YES |
| 22 | [Df(3L)ED4543](http://flybase.org/reports/FBab0035791.html) | 70C6;70F4 | 3L:13928325;14751140 | YES |
| 23 | [Df(3L)ED217](http://flybase.org/reports/FBab0035331.html) | 70F4;71E1 | 3L:14751170;15582196 | YES |
| 24 | [Df(3L)Exel6127](http://flybase.org/reports/FBab0038147.html) | 72D1; 72D9 | 3L:16040040;16122754 | YES |
| 25 | [Df(3L)ED4606](http://flybase.org/reports/FBab0035850.html) | 72D4;73C4 | 3L:16080584;16773223 | YES |
| 26 | [Df(3L)ED223](http://flybase.org/reports/FBab0035335.html) | 73A1;73D5 | 3L:16444925;16883977 | YES |
| 27 | [Df(3L)ED4674](http://flybase.org/reports/FBab0035917.html) | 73B5;73E5 | 3L:16654384;17042518 | YES |
| 28 | [Df(3L)ED4685](http://flybase.org/reports/FBab0035928.html) | 73D5;74E2 | 3L:16884176;17605270 | YES |
| 29 | [Df(3L)ED4710](http://flybase.org/reports/FBab0035953.html) | 74D1;75B11 | 3L:17480563;18132399 | YES |
| 30 | [Df(3L)ED224](http://flybase.org/reports/FBab0035336.html) | 75B1;75C6 | 3L:17962303;18391619 | YES |
| 31 | [Df(3L)ED225](http://flybase.org/reports/FBab0035337.html) | 75C1;75D4 | 3L:18179245;18614437 | YES |
| 32 | [Df(3L)ED4782](http://flybase.org/reports/FBab0036023.html) | 75F2;76A1 | 3L:18988994;19163802 | YES |
| 33 | [Df(3L)ED4786](http://flybase.org/reports/FBab0036027.html) | 75F7;76A5 | 3L:19094051;19288762 | YES |
| 34 | [Df(3L)ED229](http://flybase.org/reports/FBab0035339.html) | 76A1;76E1 | 3L:19163806;19995811 | YES |
| 35 | [Df(3L)ED4858](http://flybase.org/reports/FBab0036097.html) | 76D3;77C1 | 3L:19888473;20394920 | YES |
| 36 | [Df(3L)Exel6136](http://flybase.org/reports/FBab0038156.html) | 77B2; 77C6 | 3L: 20303330; 20486308 | YES |
| 37 | [Df(3L)ED4978](http://flybase.org/reports/FBab0036217.html) | 78D5;79A2 | 3L:21526907;21873785 | YES |
| 38 | [Df(3L)ED230](http://flybase.org/reports/FBab0035340.html) | 79C2;80A4 | 3L:22127751;22827471 | YES |
| 39 | [Df(3L)ED5017](http://flybase.org/reports/FBab0036254.html) | 80A4;80C2 | 3L:22828597;22991401 | YES |
| 40 | [Df(3R)ED](http://flybase.org/reports/FBab0043840.html)*5100* | 81F6;82E7 | 3R:22995;912807 | YES |
| 41 | [Df(3R)ED5142](http://flybase.org/reports/FBab0036381.html) | 82B2;82F8 | 3R:279018;1090605 | YES |
| 42 | [Df(3R)Exel6144](http://flybase.org/reports/FBab0038199.html) | 83A6; 83B6 | 3R:1328532;1438438143 | YES |
| 43 | [Df(3R)ED10257](http://flybase.org/reports/FBab0043840.html) | 83A7;83B4 | 3R:1344078;1426000 | YES |
| 44 | [Df(3R)ED5197](http://flybase.org/reports/FBab0036436.html) | 83B7;83D2 | 3R:1474504;1833866 | YES |
| 45 | [Df(3R)ED7665](http://flybase.org/reports/FBab0037582.html) | 84B4;84E11 | 3R:2916249;3919805 | YES |
| 46 | [Df(3R)ED5230](http://flybase.org/reports/FBab0036469.html) | 84E6;85A5 | 3R:3803496;4478856 | YES |
| 47 | [Df(3R)ED5330](http://flybase.org/reports/FBab0036566.html) | 85A5;85D1 | 3R:4495308;5055517 | YES |
| 48 | [Df(3R)ED5339](http://flybase.org/reports/FBab0036575.html) | 85D1;85D11 | 3R:5052798;5178097 | YES |
| 49 | [Df(3R)ED5454](http://flybase.org/reports/FBab0036690.html) | 85E5;85F12 | 3R:5552399;5937180 | YES |
| 50 | [Df(3R)ED5495](http://flybase.org/reports/FBab0036731.html) | 85F16;86C7 | 3R:5996223;6712482 | YES |
| 51 | [Df(3R)ED5559](http://flybase.org/reports/FBab0036790.html) | 86E11;87B11 | 3R:7394904;8269738 | YES |
| 52 | [Df(3R)ED5577](http://flybase.org/reports/FBab0036807.html) | 86F9;87B13 | 3R:7654463;8303300 | YES |
| 53 | [Df(3R)ED5591](http://flybase.org/reports/FBab0036821.html) | 87B7;87C7 | 3R:8176253;8545732 | YES |
| 54 | [Df(3R)ED5623](http://flybase.org/reports/FBab0036852.html) | 87E3;88A4 | 3R:9085471;9809634 | YES |
| 55 | [Df(3R)ED5642](http://flybase.org/reports/FBab0036871.html) | 87F10;88C2 | 3R:9509544;10307496 | YES |
| 56 | [Df(3R)ED5644](http://flybase.org/reports/FBab0036873.html) | 88A4;88C9 | 3R:9843625;10451431 | YES |
| 57 | [Df(3R)ED](http://flybase.org/reports/FBab0037009.html)*10639* | 89B7;89B18 | 3R:12038635;12306942 | YES |
| 58 | [Df(3R)ED10642](http://flybase.org/reports/FBab0044185.html) | 89B17;89D5 | 3R:12279479;12450993 | YES |
| 59 | [Df(3R)ED5780](http://flybase.org/reports/FBab0037009.html) | 89E11;90C1 | 3R:12882199;13507523 | YES |
| 60 | [Df(3R)ED5797](http://flybase.org/reports/FBab0037026.html) | 90C2;90F10 | 3R:13543832;14068391 | YES |
| 61 | [Df(3R)ED2](http://flybase.org/reports/FBab0029731.html) | 91A5;91F1 | 3R:14224953;14922493 | YES |
| 62 | [Df(3R)ED5911](http://flybase.org/reports/FBab0037139.html) | 91C5;91F4 | 3R:14568649;14991505 | YES |
| 63 | [Df(3R)ED](http://flybase.org/reports/FBab0037139.html)*6025* | 92A11;92E2 | 3R:15468450;16135241 | YES |
| 64 | *Df(3R)e^gp4^* | 93B11-13;93D6-7 | NA | YES |
| 65 | [Df(3R)e-H4](http://flybase.org/.bin/fbidq.html?FBab0002778) | 93D1; 93F6-8 | NA | YES |
| 66 | *Df(3R)GC14* | 93D6-7; 93D10 | NA | YES |
| 67 | [Df(3R)ED6085](http://flybase.org/reports/FBab0037306.html) | 93F14;94B5 | 3R:17706717;18413461 | YES |
| 68 | [Df(3R)ED6096](http://flybase.org/reports/FBab0037317.html) | 94B5;94E7 | 3R:18413403;19047691 | YES |
| 69 | [Df(3R)ED6187](http://flybase.org/reports/FBab0037407.html) | 95D10;96A7 | 3R:19877370;20369665 | YES |
| 70 | [Df(3R)ED6220](http://flybase.org/reports/FBab0037440.html) | 96A7;96C3 | 3R:20369520;21009495 | YES |
| 71 | [Df(3R)ED6235](http://flybase.org/reports/FBab0037455.html) | 97B9;97D12 | 3R:22360956;22806229 | YES |
| 72 | [Df(3R)ED6265](http://flybase.org/reports/FBab0037485.html) | 97E2;98A7 | 3R:22937981;23405492 | YES |
| 73 | [Df(3R)ED6310](http://flybase.org/reports/FBab0037530.html) | 98F12;99B2 | 3R:24964617;25337875 | YES |
| 74 | [Df(3R)ED6316](http://flybase.org/reports/FBab0037536.html) | 99A5;99C1 | 3R:25081045;25608389 | YES |
