# Supplementary material for: Drosophila Dynein Intermediate Chain Gene, Dic61B, Is Required for Spermatogenesis
Source: PLoS One. 2011 Dec 1;6(12):e27822. doi: 10.1371/journal.pone.0027822 (PMC3228723; doi:10.1371/journal.pone.0027822)
Supplement: Table S2 — Transposon insertion lines tested for complementation with the ms21 allele. Stocks obtained from Bloomington and Exelixis stock centers are shown in tables S2A and S2B, respectively. (DOC) [file pone.0027822.s004.doc]

**Table S**2A

| **S. No.** | **Stock**  **#** | **Symbol** | **Estimated Cytology *** | **Genomic sequence Coordinates *** | **Complementation** |
| --- | --- | --- | --- | --- | --- |
| 1 | 21615 | [P{wHy}DG06107](http://flybase.org/reports/FBti0074755.html) | 61B1 | 3L:122938..122938 | YES |
| 2 | 22972 | p130CAS^MB01039a^ | 61B1-B2 | 3L:161150..161150 | YES |
| 3 | 24711 | p130CAS^MB04490^ | 61B1-B2 | 3L:172063..172063 | YES |
| 4 | 21829 | [P{Mae-UAS.6.11} CG7049[DP00954]](http://flybase.org/reports/FBti0074768.html) | 61B2 | 3L: 156856..156856 | YES |
| 5 | [10628](http://flystocks.bio.indiana.edu/Reports/10628.html) | [P{ry11}E(bx)[ry122]](http://flybase.org/reports/FBti0025342.html) | 61B2-B3 | 3L:234101..246901 | YES |
| 6 | [14503](http://flystocks.bio.indiana.edu/Reports/14503.html) | [P{SUPor-P}mRpL17 [KG06809]](http://flybase.org/reports/FBti0024588.html) | 61B3 | 3L:265003..265003 | YES |
| 7 | [16303](http://flystocks.bio.indiana.edu/Reports/16303.html) | [PBac{3HPy[+]}C205](http://flybase.org/reports/FBti0037877.html) | 61B3 | 3L:275156..275156 | YES |
| 8 | [17101](http://flystocks.bio.indiana.edu/Reports/17101.html) | [P{EP}EP3223 [EP3223]](http://flybase.org/reports/FBti0011396.html) | 61C1 | 3L: 319907..319907 | YES |
| 9 | [17137](http://flystocks.bio.indiana.edu/Reports/17137.html) | [P{EP}EP3542](http://flybase.org/reports/FBti0011651.html) | 61C1 | 3L:329270..329270 | YES |
| 10 | [18405](http://flystocks.bio.indiana.edu/Reports/18405.html) | [PBac{WH}Ptpmeg [f01047]](http://flybase.org/reports/FBti0041972.html) | 61C1 | 3L:334377..334377 | YES |
| 11 | [17362](http://flystocks.bio.indiana.edu/Reports/17362.html) | [P{EPgy2}CG1233[EY06504]](http://flybase.org/reports/FBti0038733.html) | 61C1 | 3L:361567..361567 | YES |
| 12 | [11747](http://flystocks.bio.indiana.edu/Reports/11747.html) | [P{PZ}trh[10512]](http://flybase.org/reports/FBti0002973.html) | 61C1 | 3L:377299..377299 | YES |
| 13 | [16204](http://flystocks.bio.indiana.edu/Reports/16204.html) | [PBac{5HPw[+]}B347](http://flybase.org/reports/FBti0037778.html) | 61C1 | 3L:315808..315808 | YES |

**Table S2B**

| **S.**  **No.** | **Line ID** | **Symbol** | **Gene** | **Cyto location *** | **Insertion site *** | **Complementation** |
| --- | --- | --- | --- | --- | --- | --- |
| 1 | d02745 | [P{XP}Pk61Cd02745](http://www.flybase.org/reports/FBti0054951.html) | Pk61C | 61B1 | 3L:131,534[+] | YES |
| 2 | e04086 | [Pbac{RB}Pk61Ce04086](http://www.flybase.org/reports/FBst1015469.html) | Pk61C | 61B1 | 3L:131685 (-) | YES |
| 3 | d04261 | [P{XP}Pk61Cd04261](http://www.flybase.org/reports/FBst1010310.html) | Pk61C | 61B1 | 3L:140940 (-) | YES |
| 4 | d00830 | [P{XP}Pk61C^d00830^](http://flybase.org/reports/FBti0054221.html) | Pk61C | 61B1 | 3L:131692 | YES |
| 5 | **c05439** | ***PBac{PB}CG7051^c05439^*** | **CG7051** | **61B1** | **3L:152445(-)** | NO |
| 6 | **f07138** | ***PBac{WH}CG7051^f07138^*** | **CG7051** | **61B1** | **3L: 151697(+)** | NO |
| 6 | e03132 | [PBac{RB}p130CAS^e03132^](http://flybase.org/reports/FBti0048112.html) | p130CAS | 61B1 | 3L:154376(+) | YES |
| 7 | c01053 | [PBac{PB}p130CAS^c01053^](http://www.flybase.org/reports/FBst1005503.html) | p130CAS | 61B2 | 3L:167,805(-) | YES |
| 8 | c00518 | [PBac{PB}p130CAS^c00158^](http://flybase.org/reports/FBti0043044.html) | CG18769 | 61B1 | 3L:6,544,504(-) | YES |
